# The low and declining risk of malaria in travellers to Latin America: is there still an indication for chemoprophylaxis?

**DOI:** 10.1186/1475-2875-6-114

**Published:** 2007-08-23

**Authors:** Ron H Behrens, Bernadette Carroll, Jiri Beran, Olivier Bouchaud, Urban Hellgren, Christoph Hatz, Tomas Jelinek, Fabrice Legros, Nikolai Mühlberger, Bjørn Myrvang, Heli Siikamäki, Leo Visser, TropNetEurop

**Affiliations:** 1 Travel Clinic, Hospital for Tropical Diseases, Mortimer Market, London, WC1E 6JB, UK; 2 London School of Hygiene & Tropical Medicine, Clinical Research Unit, London, UK; 3 University Hospital, Department of Infectious Diseases, Hradec Králové, Czech Republic; 4 Hôpital Avicenne, Department of Infectious and Tropical Diseases, Bobigny, France; 5 Karolinska Institute, Huddinge University Hospital, Division of Infectious Diseases, Stockholm, Sweden; 6 Swiss Tropical Institute, Basel, Switzerland; 7 Berlin Centre for Travel and Tropical Medicine, Berlin, Germany; 8 Centre National de Référence du Paludisme, Paris, France; 9 Institute for Public Health, University for Health Sciences, Medical Informatics and Technology, Eduard Wallnöfer-Zentrum I, A-6060 Hall i.T., Austria; 10 Ullevaal University Hospital, Department of Infectious Diseases, Oslo, Norway; 11 Helsinki University Central Hospital, Department of Medicine, Division of Infectious Diseases, Helsinki, Finland; 12 Leiden University Medical Centre, Department of Infectious Disease, Section Travel Medicine, Leiden, The Netherlands

## Abstract

A comparison was made between local malaria transmission and malaria imported by travellers to identify the utility of national and regional annual parasite index (API) in predicting malaria risk and its value in generating recommendations on malaria prophylaxis for travellers.

Regional malaria transmission data was correlated with malaria acquired in Latin America and imported into the USA and nine European countries. Between 2000 and 2004, most countries reported declining malaria transmission. Highest API's in 2003/4 were in Surinam (287.4) Guyana (209.2) and French Guiana (147.4). The major source of travel associated malaria was Honduras, French Guiana, Guatemala, Mexico and Ecuador. During 2004 there were 6.3 million visits from the ten study countries and in 2005, 209 cases of malaria of which 22 (11%) were *Plasmodium falciparum*. The risk of adverse events are high and the benefit of avoided benign vivax malaria is very low under current policy, which may be causing more harm than benefit.

## Background

Many public health bodies base their recommendations for the prevention of malaria in travellers on national surveillance data, which provides information on the intensity and risk of malaria in local populations, expressed as the annual parasite index (API), which may reflect regional risks. While this approach appears rational, there is no evidence that patterns of travel-acquired malaria correlate with transmission intensity among indigenous populations. Recommendations on prophylaxis for travellers need to balance the threat of malaria, including falciparum malaria, and the risk of a fatal outcome, against the potential toxicity of chemoprophylaxis, a risk which is not relevant to populations living in endemic regions. Rombo called into question the use of API to estimate the risk of travellers acquiring malaria [1]. Highlighting the disparity between native and traveller's vulnerability and exposure to infection he has emphasized the need to consider prophylaxis toxicity when prescribing for travel to low risk malaria regions.

Providing appropriate malaria prophylaxis advice for travellers visiting countries in Central and South America can be complex and challenging, particularly when the journey involves many regions or countries where multiple parasite species are present.

This study was set up to review rates of malaria transmission within Central and South American countries and to compare these with patterns of imported malaria among European and US travellers returned from endemic countries. The aim of the study was to try and identify whether transmission within a country reflects malaria transmission among travellers and to examine the usefulness of API in predicting travellers' risk and its value as a basis for recommendations of malaria prophylaxis.

## Methods

The local population risk is based on reports from the Pan American Health Organization (PAHO) Regional Office of the World Health Organization with information on API provided by countries within the region[2,3]. The change in the API over the period 1998 and 2004 has been included to reflect changing trends of malaria transmission. All countries provide regional and district API data, and for this study the highest API in each country is used to represent the maximum risk likely to be faced by travellers. The malaria risk associated with travel is identified through reports from National malaria surveillance bodies, describing malaria imported in returned travellers from Central and South America (Table 1) between the years 2000 and 2005 (data for 2005 was not available for France) from nine European countries and the USA (Table 1). Most of this data was provided through and from members of TropNetEurop, a network of clinical sites, which have access to national malaria surveillance reports, and from the literature. Most reports do not contain details on region of travel within countries, and where several countries have been visited, reports do not necessarily reflect the country of acquisition. Cases provided by TropNetEurop were not included in the country malaria analysis as they would duplicate case reports. Malaria imported from Mexico was analysed separately from the Central America region.

**Table 1:** Regions and Countries from Central and South America included in the destination analysis and countries reporting imported malaria.

**Central America**	**South America**
Belize	Argentina
Costa Rica	Bolivia
El Salvador	Brazil
Guatemala	Colombia
Honduras	Ecuador
Nicaragua	French Guiana
Panama	Guyana
	Paraguay
Mexico	Peru
	Surinam
	Venezuela
C America unspecified	S America unspecified

**Reported imported Malaria**	

Czech Republic	Finland
France	Germany
Holland	Norway
Sweden	Switzerland
United Kingdom	United States of America

**Figure 1:**
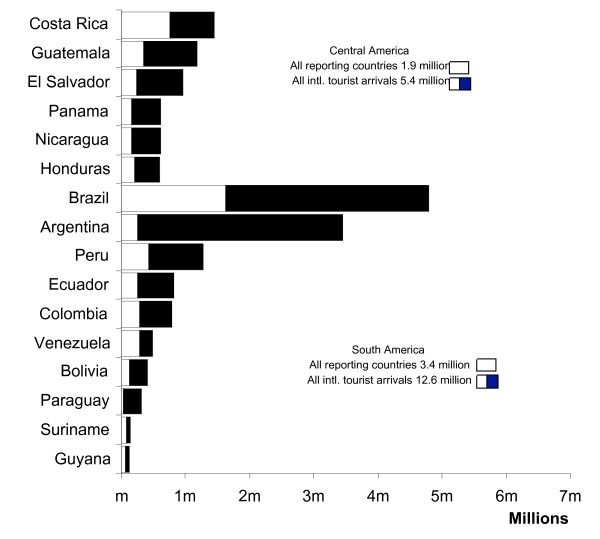
Total visits made by international tourists to the study countries adjusted to visits from the reporting countries.

The volume of travel will have a significant bearing on the number of cases of imported malaria and therefore rates, where possible, were calculated. The World Tourism Organization collects data on international arrivals and this data provides an estimate of the number of tourist departures and arrivals by country. This data was used where no national statistics were available to estimate the numbers of visits made from the study countries [4,5] (Figure 1). Malaria cases recorded in UK travellers were analysed using a denominator, the number of visits made by UK citizens to the countries of malaria acquisition. Data provided by the International Passenger Survey (IPS) is collected through face-to-face interviews of passengers at all major ports within the UK. A quarter of a million passengers are interviewed throughout the year and this sample provides an estimate of the total annual visits to each country, the duration of stay and reason for travel. Malaria cases occurring in United States travellers were extracted from data published by the Centers for Disease Control [6-11] and visits made by US citizens to the region were collected by the USA International Air Travel Statistics (or I-92) programme. This provides data on outbound numbers of US citizens travelling, using point-to-point air traffic totals from the USA, on departing flights. Visits to, and malaria from Mexico were analysed separately due to the large travelling population from the USA[12]. Malaria data from France was provided through a reporting network of 120 selected hospital laboratories covering approximately half of annual estimates of malaria cases to the Malaria National Reference Centre (CNRPalu). French and Dutch denominators were captured using a methodology similar to that of the USA International Air Travel Statistics (I-92) programme, which reflects aircraft coupons capturing passengers departing to specific destinations between 2000 and 2004. The data on denominators were provided by the French Aviation Authority and Statistics Netherlands, but capture methods vary. In the analysis, malaria acquired in Mexico and visits to Mexico were excluded from the Central America groupings.

## Results

### Malaria risk in Central and South America

Within the region, 21 countries reported malaria transmission [13,3]. An estimated 264 million out of the 867 million inhabitants were at risk of malaria, 11 million of these at high risk [3]. Between 1998 and 2004 in countries popular with travellers, Brazil, Peru, Ecuador and Colombia in South America, and Guatemala and Honduras in Central America, there was a decline in both annual positive slides and API in high risk regions in all but Colombia and Honduras (Table 2), and a decline in the absolute number of cases in all countries except for Peru and Colombia[14]. The most recent API's ranged from 0.07 to 287 in the highest risk regions. Mexico reported low transmission in two provinces only [3].

**Table 2:** Numbers of malaria risk regions in popular tourist destinations, reflecting changing incidence, highest risk regions and species diagnosed during surveillance

		**Average API's in Moderate and High risk regions**				**2004§**			
					
**Country**	**Highest risk regions with total provinces/departments‡**	**2004#**	**1998**	**% change API 1998–2004**	**Highest regional API 2004¥**	**P.F.**	**P.V.**	**Totals**	**P.V.%**
Guatemala	4/26	9.6	15.8	-39%	53.68 (Peten Sur Occidente)	1,300	28,983	30,283	96%
Honduras	5/9	12.6	9.2	36%	26.55 (Islas de la Bahia)	283	9,033	9,316	97%
Brazil	66/5561	28.0	64.0	-56%	242.05 (Tocantins)	75,685	276,021	351,706	78%
Colombia	18/33	26.2	12.1	116%	233.92 (Cordoba)	42,633	69,272	111,905	62%
Ecuador	12/22	12.0	15.2	-21%	64.43 (Quininde)	5,891	22,839	28,730	79%
French Guiana	5/5	147.4	216.4	-32%	231.27 (Maripasuola)	1,901	752	2,653	28%
Peru	12/34	11.7	21.6	-46%	112.60 (Tumbes)	14,740	74,720	89,460	84%
Surinam	6/10	287.39	263.96	9%	686.07 (Upper Saramacca)	12,078	1,494	13,572	11%
Mexico	2/32	0.07	0.44	-84%	0.30 (Oaxaca)	49	3,357	3,406	99%

‡Data for Colombia, Ecuador, French Guiana, Suriname, & Honduras is taken from PAHO Malaria programs in the America (based on 2002 data), the remaining data is taken from Malaria programs in the Americas (based on 2004 data).

#Data for Brazil, French Guiana & Surinam is 2003 data

¥Data for Colombia & Suriname is taken from PAHO Malaria programs in the America (based on 2002 data), the remaining data is taken from Malaria programs in the Americas (based on 2004 data).

§Data from 2003

### Imported malaria

**Figure 2:**
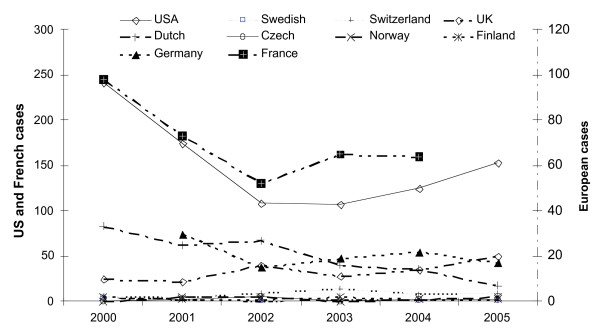
Imported cases by reporting countries 2000–2005. Y axis 1 reflects US and French cases, all other countries are shown against Y axis 2.

Case reports from Central and South America constitute a small proportion of total imported malaria. In the USA, they accounted for 10% of the total imported malaria in 2005, whilst in Europe, in the same year, the proportion ranged from 1.1% in the UK, 2% (2004) in France, 3.4% in Switzerland and 2.4% in the Netherlands. By species, *Plasmodium falciparum *infections ranged between 3% and 17% of all malaria reports annually from Central America and between 17% and 24% of cases acquired in South America. The total number of imported malaria cases reported to surveillance bodies annually for the years 2000 to 2005 inclusive, fell from 395 to 209 cases of which 69% were non falciparum in 2005. The bulk of cases were reported in American travellers, with Guatemala and Honduras being the main sources of infection. Total USA reports fell from 242 to 153 in 2005 of which 84% were non-falciparum, where the species was known. Six countries reported 10 or less imported malaria cases from Central and South America in 2005 (Figure 2).

**Figure 3:**
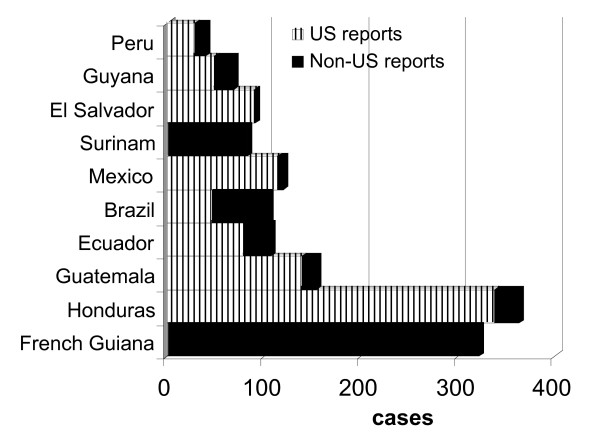
Total imported malaria from 9 countries stratified by US and Non US reporting countries 2000–2005.

Guatemala, French Guiana and Honduras provided half (55%) of all imported malaria over the 5 years. French Guiana was an important source of malaria for French travellers and Surinam for Dutch travellers. Surinam is a popular destination for Dutch travellers, (60% of all international arrivals in 2004 were from the Netherlands [15] and it is the source of 60% (37% *P. falciparum*) of all malaria cases in Dutch travellers from Latin America. Eighty nine percent of all malaria in French travellers was acquired in French Guiana, where they make up approximately two thirds of all tourist arrivals. Seventy percent were *Plasmodium vivax *infections and twice as many cases occurred in civil as in military personnel, although a high incidence has been reported in the military [16]. The rate in French residents returned from French Guiana averaged 6.2 per 10,000 visits (*P. falciparum *1.3/10,000 and *P. vivax *4.3/10,000). Honduras accounted for the largest source of infection, most were in US travellers (Figure 3) who made up over a quarter (178,285) of all tourists arrivals in 2004. Cases from Honduras, predominantly in US travellers have declined by 20% between 2000 and 2005, despite the API having increased by 36% in indigenous populations (Table 2). The rate in Honduras was the highest for all countries visited by UK travellers (5.6/10,000). There were five countries (Honduras, Nicaragua, Surinam, French Guiana and Guatemala) where the rate of malaria was >1/10,000 visits for UK travellers. Data on the duration of visit was available for visits by UK travellers, a case per years travelling (proxy of exposure) was calculated, based on total nights away, visitors and numbers of cases of malaria. The average duration of visit by UK travellers to the South American continent (2005) was 18 days. Mexico had the lowest risk where one case occurred for very 22,664 years exposed. The risks for Peru, Columbia and Brazil were similar, around one case for every 3,000 years exposed, and similar to the risks of UK residents visiting India [17]. Honduras and Guatemala were the highest risk countries with one case for every 103 and 513 years exposed respectively.

Low malaria rates were also noted in UK, French and Dutch visitors to Venezuela, Colombia, Peru and Brazil during 2004. These four countries received a total 2.6 million visitors from the study countries (Figure 1) and had a maximum incidence of malaria of 2.2 (UK travellers to Colombia, 2004) per 10,000 visits.

## Discussion

This study was designed to identify whether local transmission of malaria within countries of Latin America reflected the pattern and trends of malaria acquired by travellers from ten developed countries. During 2004, twenty one PAHO countries with active malaria programs examined 6.7 million slides, of which 13% were positive for malaria, three quarters of them were speciated as *P. vivax *infections in an "at risk" population of 262 million [3]. The detailed information collected in Central and South American countries and presented by PAHO [3,2,13] provides evidence of a clear trend of declining transmission across most of the countries, most notably in Brazil, which reported a 56% decrease in the high incidence regions, attributed to malaria control programmes initiated in 2000. The total number of tourists visiting Latin America is not known precisely, but the World Tourism Organization [4] estimates there were 16 million international tourist arrivals to South America, with a 16% increase from 2003. Central America, during 2004, received 5.7 million inbound visitors, a 17% growth in arrivals over the previous year. The main country sources of imported malaria were Honduras, French Guiana, Guatemala, Mexico and Ecuador, from where there were 1,066 imported cases over five years, accounting for 64% of all imported cases from Latin America, 75% were non-falciparum malaria. There are a number of important limitations that need to be understood when reflecting on the findings. Local transmission reported to PAHO may be inconsistent and regions not reporting or not diagnosing cases may be interpreted as no malaria transmission. The imported malaria cases collected nationally use different reporting methods and are of varied quality. The denominators used in the analysis are again of different capture methods. The USA, France and the Netherlands record the number of citizens departing to a destination while in the UK samples of departing passengers are interviewed, capturing destination, duration of travel and reason for travel. The pattern of travel through the regions by western travellers is not recorded in the denominator data or through the malaria case reports, and therefore the proportion visiting high transmission regions are unknown.

Although a number of regions within Peru and Brazil have an API above 50/1000 cases/year the actual numbers of malaria cases in returning travellers is low, a total of 145 cases over five years, and in 2005, there were only 30 cases of which three were *P. falciparum*. Asymptomatic carriage in natives living in the Peruvian Amazon near Iquitos is estimated to be less than 10% and the entomological inoculation rate for the Amazonas region reported as 10–20 annually [18]. The small numbers of travel associated cases from Peru are unlikely to be a result of widespread use of chemoprophylaxis. Currie and colleagues [19] examined prophylaxis use in tourists departing Lima, Peru. Of the 1226 travellers interviewed, 43% were from the USA. Nearly three quarters had visited only Peru and 54% had visited a malarious region (as defined by CDC). Of these around half had taken regular chemoprophylaxis (42% atovaquone/proguanil). During that year (2003) there were 10 (six *P vivax*) imported malaria cases from Peru. The highest numbers of imported malaria cases, over the 5-year study period, were of *P. vivax *from Honduras, Guatemala, Ecuador and French Guiana. Despite an increase in local transmission in Honduras, total travel associated cases declined by 20% suggesting that there is no correlation between the two trends. The rates in US and UK travellers to the whole region (excluding Mexico) reveal a similar incidence of 0.3 and 0.8 per 10,000 visits despite an increasing volume of travel over the study (237,526 UK and 4.5 million US travellers in 2005).

Mexico had an estimated 20 million visits by US citizens in 2004. Visits to malaria endemic regions of Mexico are unknown, but are likely to be small. There was a fall in imported malaria to the U.S. from Mexico, from 30 case reports in 2000 to 14 in 2005.

Current chemoprophylaxis policies recommend prophylaxis for high risk regions [20-22], but many of these regions (as shown in Table 2 have a declining risk for indigenous populations). The inconsistency between focal high transmission areas in countries popular with western travellers and small numbers of travel associated malaria is worth exploring. Significant numbers of travellers may not be using prophylaxis during their travel and the departure lounge suggests approximately 50% of visitors will be using chemoprophylaxis. Other countries visited by significant numbers of tourists as reported by WTO in 2005 – Peru, (1.5 million) Brazil (5.4 million), Guatemala (1.3 million) had small numbers of cases and low rates of malaria. Although these countries have areas of high transmission, the major parts of these countries have no malaria transmission. It would appear that most visitors to these countries are at low or no risk of acquiring infection, whatever their journey and destination within the country.

Protection against *P. vivax*, disease despite using the most widely available regimens is marginal [23-25], as only the primary attack [23] is aborted. Most clinical episodes develop some months after infection when travellers have returned home and are unlikely to be missed through routine reporting systems. Severe adverse events leading to stopping medication during chemoprophylactic drug use were reported in 3–8% of users whilst mild to moderate adverse events were reported by 32%–45% of users [26]. In the 423,416 visitors from reporting countries to Peru in 2003 [4] approximately 25% (105,000 or 50% of those visiting a malarious region) visitors were using chemoprophylaxis as identified by the airport departure lounge study [19]. During that year, 10 (two *P. falciparum*) cases of malaria were reported in nine study countries after visiting Peru. Using the minimal proportion of users encountering adverse events from the popular prophylaxis regimens [26] an estimated 34,000 travellers would have suffered an adverse event related to chemoprophylaxis use. The risk of adverse events for visitors to Peru and other regions are likely to be significantly higher than avoided infections particularly of benign *P. vivax *malaria under current policy recommendations. Unless chemoprophylaxis prescribing is significantly reduced, current recommendations are likely to be causing more harm than benefit.

### Policy change

Despite its limitations, this study suggests that the risk of adverse events from chemoprophylaxis is likely to be significantly higher than the risk of acquiring malaria in the most popular tourist destinations in Central and South America. Although current national and international policy focuses on chemoprophylaxis for focal, highly endemic malaria transmission regions in countries which have overall low API's, this strategy appears to provide limited benefit as travellers appear to have a low malaria attack rate and will acquire *P. vivax *rather than *P. falciparum *infection. The benefit of chemoprophylaxis in preventing the former is unclear. An alternate strategy adopted by a number of European countries, for example Switzerland [27], is to provide travellers with emergency standby treatment in case of malaria symptoms during travel. This has the benefit of dealing with a life threatening attack of falciparum malaria, but avoiding adverse events associated with excessive chemoprophylaxis. It has the disadvantage of cost, as all travellers will have to purchase therapy. Two of the highest risk countries reported by PAHO – French Guiana, and Surinam, correlated to countries where visitors were at high risk of malaria and chemoprophylaxis would be appropriate for travel to risk areas in these countries. There appears to be no clear benefit and significant potential for toxicity in recommending chemoprophylaxis for visitors to Mexico, where the highest API is less than 0.07 for local residents and 20 imported cases annually. Despite the low or falling risk of malaria, the continued use of bite prevention measures remains important as these are effective, safe and have the added benefit of reducing other vector borne diseases.

## Authors' contributions

RHB and BC designed the study, collated the data and prepared the first draft.

JB, OB, UH, CH, TJ, FL, NM, BM, HS AND LV obtained and analysed national data. All authors contributed to the interpretation of the data and agreed the final draft.

## Data sources

USA denominator data: In flight survey ITA Office of Travel & Tourism Industries.

UK cases: Malaria Reference Laboratory, Health Protection Agency UK (Peter Chiodini).

UK denominator data: IPS Office for National Statistics.

France: Centre National de Référence du Paludisme.

TropNetEurop 

Finland: National Public Health Institute, Finland.

Jorge Atouguia, Instituto de Higiene e Medicina Tropical, Lisboa.

Czech Republic National Institute of Public Health in Prague,: C. Benes.

Switzerland: Simone Graf (Swiss Federal Office of Public Health).

Netherlands: National Center of Disease Control Institute for Public Health and the Environment Norway: Section of Infectious Disease Prevention and Control, the Norwegian Institute of Public Health (Hans Blystad).
